# Ophiopogonin B alleviates cisplatin resistance of lung cancer cells by inducing Caspase-1/GSDMD dependent pyroptosis

**DOI:** 10.7150/jca.66432

**Published:** 2022-01-01

**Authors:** Ziyu Cheng, Zhihui Li, Ling Gu, Liqiu Li, Qian Gao, Xiongfei Zhang, Jin Fu, Yuanyuan Guo, Qirui Li, Xu Shen, Meijuan Chen, Xu Zhang

**Affiliations:** 1 School of Medicine & Holistic Integrative Medicine, Nanjing University of Chinese Medicine, Nanjing 210023, People's Republic of China.; 2 College of Traditional Chinese Medicine & Integrated Chinese and Western Medicine College, Nanjing University of Chinese Medicine, Nanjing 210023, People's Republic of China.

**Keywords:** non-small cell lung cancer (NSCLC) cells, drug resistant, ophiopogonin B (OP-B), pyroptosis, Caspase-1/GSDMD

## Abstract

Drug resistance has become the main reason for the failure of tumor chemotherapy. *Radix Ophiopogon Japonicus* has long been used as traditional Chinese medicine to treat pulmonary disease, and Ophiopogonin B (OP-B) as a bioactive component of it has also been verified to inhibit cell proliferation of various non-small cell lung cancer (NSCLC) cells *in vivo* and *in vitro*. Therefore, we wonder whether OP-B is also effective to drug resistant lung cancer cells. Firstly, Cell Counting Kit-8 (CCK8) assay was used to compare the sensitivity of OP-B on NCI-H460, A549, cisplatin resistant A549 (A549/DDP) and paclitaxel resistant A549 (A549/PTX) cells, and A549/DDP cells were shown to be more sensitive to OP-B than other three cell lines, the results were further verified in orthotopic tumor nude mice model and zebrafish tumor model. Moreover, observation of cell morphological feature, mitochondrial membrane potential, LDH release rate, and production of IL-1β all suggested that OP-B induced pyroptosis in A549/DDP cells more significantly than that in A549 cells. Meanwhile, transcriptomic sequencing results between OP-B treated and the Mock A549/DDP group also suggested that OP-B induced more significant Caspase-1/GSDMD dependent pyroptosis in A549/DDP group, which was further verified by VX-765, the inhibitor of Caspase-1. Together, the experimental results suggested that OP-B alleviated DDP resistance of A549 cells through inducing more significant Caspase-1/GSDMD-dependent pyroptosis.

## Introduction

In recent years, lung cancer has become the third leading cause of cancer-related diseases 1. The clinical classification of lung cancer is complex and diverse, while NSCLC accounts for up to 85% of the patients 2. Currently, chemotherapy remains the standard of treatment for NSCLC patients due to approximately 85-90% NSCLC having no driver mutations defined for drug target 3. However, with the long-term application of basic chemotherapy drugs in clinical practice, the problem of tumor drug resistance becomes increasingly serious, which has been the main reason for the inefficiency of tumor chemotherapy 4. Therefore, it's very urgent to develop new drugs or find sensitizers for them to improve clinical efficacy.

The mechanism of drug resistance in tumors are complex, which mainly includes drug concentration reduction in tumor cells, abnormal drug metabolic pathway, DNA damage repair dysfunction, regulation of autophagy, blocking of apoptotic pathways, and self-renewal or proliferation of tumor stem cells 5-8. Pyroptosis is a type of programmed cell death that associated with inflammation, which characterized by cell swelling, destruction of membrane structural integrity and cytoplasmic contents release, like interleukin-1β (IL-1β), interleukin-18 (IL-18) and lactate dehydrogenase (LDH) 9. The activation of classical and non-classical pathway is separately dependent on Caspase-1 or Caspase-4/5/11 10, 11. Activated Caspase-1/4/5/11 catalyze GSDMD to cleave into GSDMD-N and GSDMD-C fragments, and GSDMD-N can bind and form pores at cell membrane, so as to lead pyroptosis and release IL-1β and IL-18 to extracellular environment 12, 13. Shi J *et al*. found that GSDMD-N itself can directly induce pyroptosis, which indicates that GSDMD cleavage is really an important index to detect whether pyroptosis occurs or not 14.

Currently, some studies found that there is any connection between tumor resistance and pyroptosis 15. For example, Wu M *et al*. combined BI2536 (an inhibitor of PLK1) with Cis-Dichlorodiammine platinum (DDP) to treat ESCC cells and found the combination of two drugs had a synergistic effect on tumor inhibition and resulted in pyroptotic death of cancer cells by Caspase-3/GSDME pathway. Their further research revealed that BI2536 can reverse DDP resistance of ESCC by inhibiting DNA damage repair and inducing pyroptosis 16.

Traditional Chinese medicine has been used for thousands of years in China and has unique advantages in cancer treatment. Ophiopogonin B (OP-B) is a bioactive component extracted from *Radix Ophiopogon Japonicus*, a traditional Chinese medicine often used in pulmonary disease treatment. In our previous studies, OP-B had been verified to have significant inhibition effects on various NSCLC cells 17-19. Herein, we focused on comparing the sensitivity of DDP-resistant A549 (A549/DDP) and A549 cell lines to OP-B. We found that A549/DDP cells were more sensitive to OP-B, and the underlying mechanism due to the induction of pyroptosis through Caspase-1/GSDMD pathway.

## Materials and methods

### Reagents

OP-B (CAS: 38971-41-4) and OP-D (CAS: 41753-55-3) were purchased from Shanghai Yuanye Bio-Technology Co., Ltd (Shanghai, China). OP-B and OP-D were dissolved in DMSO as a 10 mmol/l stock solution and stored at 4 °C. Caspase-1 inhibitor VX765 (Belnacasan) (CAS: 273404-37-8) were purchased from Med Chem Express. Polo-188 (CAS:9003-11-6) was purchased from Beijing solarbio science & technology co., Ltd.

### Cell culture

A549 and NCI-H460 cells were obtained from the Stem Cell Bank, Chinese Academy of Sciences (Shanghai, China). A549/DDP and A549/PTX cells were kind gifts from Professor Zhigang Guo (NanJing Normal University). A549, NCI-H460 cells were cultured in DMEM/F12 medium (Gibco, Australia), and A549/DDP, A549/PTX cells were cultured in RPMI 1640 medium (Gibco, Australia) with 10% fetal bovine serum (FBS; Gibco, Australia), supplemented with 1% penicillin/streptomycin solution (Gibco, Australia). All of the cells were maintained at 37 °C in humidified atmosphere of 5% CO_2_.

### Cell viability assays

The inhibition of OP-B on A549, NCI-H460, A549/PTX, A549/DDP cells was estimated by Cell Counting Kit-8 (CCK-8; Dojindo, Beijing, China) as described previously 20.

### Western blot analysis

The cells were lysed in radioimmunoprecipitation assay (RIPA) buffer (Beyotime, Shanghai, China) containing 1% phenylmethanesulfonyl fluoride (PMSF) before suspended in sodium dodecyl sulfate-polyacrylamide gel electrophoresis (SDS-PAGE) sample loading buffer (Beyotime, Shanghai, China), then separated on 12% SDS-PAGE (Beyotime, Shanghai, China) and transferred onto polyvinylidene fluoride (PVDF) membranes (Thermo Fisher, US). After the membranes were blocked with 5% non-fat milk, they were incubated at 4 °C overnight with primary antibodies against Caspase-1 (1:500, CST, US), GSDMD (1:500, CST, US), GSDMD-N (1:500, CST, US), NLRP3 (1:1000, CST, US), and β-actin (1:2000, CST, US). After incubation with horseradish peroxidase (HRP)-linked anti-rabbit (1:2000, CST, US) or anti-mouse (1:2000, CST, US) secondary antibodies for 2 h at room temperature, the bands were detected with a Gel Doc™ XR+ Gel Documentation System (Bio-Rad, US) with enhanced chemiluminescence (ECL) reagents (Bio-Rad, US).

### Transcriptomic sequencing

Cells were pelleted, and RNA samples were isolated and sent to Huada (Bgi Genomics Co., Ltd, China) for constructing RNA-seq library. Briefly, total RNAs were isolated, and mRNA was enriched and then were pooled together for cDNA synthesis and sequencing. The transcriptomic RNA sequencing was performed on an Illumina NovaSeq 6000 platform, to create pair-end reads with a length of 150 bp (PE150). The differentially expressed genes (DEGs) were identified using edgeR (|logFC | >1 & FDR < 0.05), and these DEGs were undergone Gene ontology and KEGG pathway analysis.

### Quantitative real-time PCR (qRT-PCR)

Total RNA was extracted from A549 or A549/DDP cells using TRIzol reagent (Sangon Biotech) according to the manufacturer's protocol. Then, the RNA was reverse transcribed to cDNA using PrimeScript™ RT reagent Kit with gDNA Eraser (Takara). Quantitative real-time PCR was performed using cDNA primers specific for mRNA. The gene GAPDH was used as an internal control. All the real-time PCR reactions were performed using Takara′s SYBR Premix Ex Taq™ II (Tli RNaseH Plus) in Applied Biosystems 7500 Fast Real-Time PCR System (Applied Biosystems). The 2^-△△Ct^ method was used for quantification and fold change for target genes was normalized by internal control.

### Immunofluorescence

Cells at a density of 1×10^4^ cells/well were seeded into 96-well plates. After exposure to the indicated treatments, the cells were fixed with 4% paraformaldehyde for 15 min at room temperature, then blocked with 5% goat serum and 0.3% Triton X-100 in phosphate-buffered saline (PBS) for 1 h. After that, the cells were incubated with a primary antibody against Cox-2 (1:200, CST, US), IL-1β (1:200, CST, US) in antibody dilution buffer (ADB; 1X PBS/1% bovine serum albumin (BSA)/0.3% Triton X-100) overnight at 4 °C. Then, the cells were incubated with a fluorochrome-conjugated anti-rabbit secondary antibody (1:1000, CST, US) in ADB for 2 h at room temperature in the dark. Subsequently, the cells were stained with DAPI (1 μg/ml, CST, US) for 5 min. Images were obtained under a fluorescence microscope (TCS SP8, Leica).

When frozen sections were used for immunofluorescence, the sections were first blocked with goat serum; the rest of the procedure was the same as that for the goat serum-blocked cells.

### LDH Release Assay

The activity of LDH released into cell culture supernatants was detected using the CytoTox 96 Non-Radioactive Cytotoxicity Assay Kit (Omega, US) according to the manufacturer's protocol for analyzing pyroptosis.

### *In vivo* xenograft assay (nude mice models and xenograft zebrafish)

The BALB/c nude mice (4 weeks old) were maintained under specific pathogen‑free (SPF) conditions. Animal welfare and experimental procedures were performed in compliance with the National Institutes of Health Guidelines for the care and use of laboratory animals, and all protocols were approved by the Ethics Review Committee of Nanjing University of Chinese Medicine. To establish the orthotopic xenograft lung cancer model, the luciferase-expressing A549 or A549/DDP cell line with lentivirus was established, then A549 or A549/DDP cells (2×10^7^ in 0.2ml medium of a 1:1 mixture of RPMI 1640 and Matrigel 354,248) were injected into right lung parenchyma of the mice (n=30), and the volume of tumors were monitored by luciferase imaging of live animals using an IVIS Spectrum bioluminescence imaging system (PerkinElmer, US) after intraperitoneal injection of 200 μl D-Luciferin substrate (15 mg/ml in DPBS, PerkinElmer). And the mice were mainly used to test the toxicity and pharmacological activity of OP-B on A549 or A549/DDP xenograft mice. The mice for toxicity-detection were divided into 5 groups (6 in each group), including polo-188 group (62.5 mg/ml Poloxamer), OP-B groups (1.5 or 3 mg/Kg OP-B), and the Normal or Mock group (saline). And the mice for pharmacological activity detection were divided into 9 groups, including the Normal group (saline), A549 or A549/DDP Mock groups (saline), A549 OP-B-treatment groups (1.5 or 3 mg/Kg OP-B), A549/DDP OP-B-treatment groups (1.5 or 3 mg/Kg OP-B), and A549 or A549/DDP cyclophosphamide groups (20 mg/Kg cyclophosphamide). All of the mice were treated with intraperitoneal injection (i.p. daily, n=28). The polo-188 was formulated with 0.9% NaCl, and OP-B was formulated with polo-188. 25 days later, all mice' hearts, livers, lungs and kidneys were harvested, then half of the tissues were used for GPT, GOT and CRE detectinon with microplate test kit (Nanjing Jiancheng Bioengineering Institute, Nanjing, China), and the rest of them were used for haematoxylin-eosin (H&E) staining, Transmission Electron Microscope (TEM) observation, or immunohistochemistry and immunofluorescence observation.

AB/wt zebrafish embryos were raised at 28 °C in fish water. At 48 hours fertilization, A549 cells, labeled with a red fluorescent dye for cell viability (Cell Tracker™ CM-DiI, Invitrogen, CA, USA) and resuspended in HBSS were injected into the yolk sac of zebrafish embryos (200 cells/embryo n=100). Then, embryos were incubated at 34 °C. At 72 h post injection, the proliferation of tumor cells was evaluated through a fluorescence stereomicroscope (OLYMPUS U-HGLGPSD, equipped with Cell Sens Entry software, Tokyo, Japan). The software Image J was used to quantify the proliferation rate of tumor cells.

### Transmission Electron Microscope (TEM) assay

The model and OP-B groups were selected for TEM analysis to observe the morphology of A549 cells and A549/DDP cells. The mice were sacrificed, 1/3 of tissue was removed from lung and fixed with 3% glutaraldehyde for more than 2 hours, then immobilized with 1% osmium acid for 2 hours. The tissues were dehydrated step by step with ethanol and acetone and then soaked overnight and embedding agent. After the tissues were embedded, polymerized, repaired, sliced and double stained with uranium acetate and lead citrate, they were observed by TEM (JEM-1011).

### Statistical analysis

Data entry and all analyses were performed in a blinded fashion. All statistical analyses were performed using Graph Pad Prism8.0 software. Statistical significance was calculated using two-tailed unpaired *t*-test on two experimental conditions or two-way ANOVA when repeated measures were compared, with *p* < 0.05 considered statistically significant. All graphs show mean values ± SEM.

## Result

### OP-B inhibited A549/DDP cell proliferation more significantly than that on A549 cells

**Figure 1:**
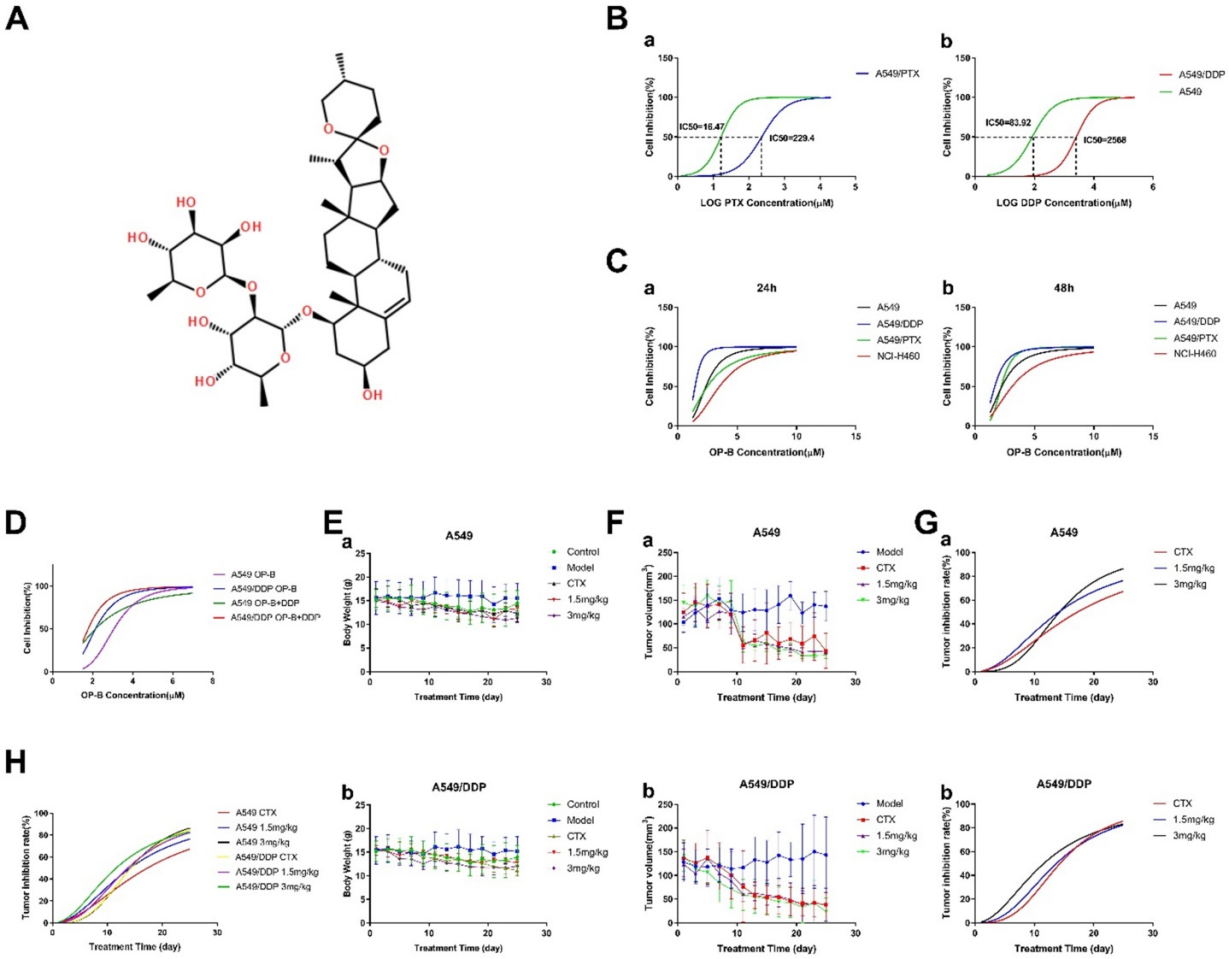
** OP-B had more significant effect on inhibiting A549/DDP cells and its transplantation tumor growth than that in A549 cells. A.** Chemical structure of OP-B. **B.** CCK8 assay tested the resistance index of A549/PTX (a) and A549/DDP (b) cells. **C.** Cell inhibition rate of NCI-H460, A549, A549/PTX and A549/DDP cells treated by OP-B for 24 (a) and 48 h (b). **D.** CCK8 assay tested inhibitory effect of OP-B combined with DDP on A549 and A549/DDP cells for 24 h. **E.** Changes of body weight in mice with A549 (a) and A549/DDP (b) transplantation tumor after treated with OP-B for 25 days. **F.** Changes of A549 (a) and A549/DDP (b) transplantation tumor volume after treated with OP-B for 25 days. **G.** Tumor inhibition rate of A549 (a) and A549/DDP (b) after treated with OP-B for 25 days. **H.** The comparison of tumor inhibition rate between A549 and A549/DDP xenograft after treated with OP-B for 25 days. The bars and error bars indicate the mean ± SD. *p < 0.05, **p < 0.01, ***p < 0.005, ****p < 0.001

First of all, we used CCK-8 assay to test cell viability of A549, A549/PTX or A549/DDP cells under different doses of PTX and DDP for 24h to prove the drug resistance of A549/DDP and A549/PTX cells (Fig. 1B). Under the treatment of PTX, the IC_50_ values of A549 and A549/PTX cells were 16.47 μM and 229.4 μM, respectively. Under the treatment of DDP, the IC_50_ values of A549 and A549/DDP cells were 83.92 μM and 2568 μM, respectively. According to the IC_50_ value of each cell, the resistance index (RI) of A549/PTX and A549/DDP cells was 13.93 and 30.6 respectively (The calculation method is: RI_(A549/PTX)_ =IC_50(A549/PTX)_/IC_50(A549)_, RI_(A549/DDP)_ =IC_50(A549/DDP)_/IC_50(A549)_).

Next, we compared the inhibitory effects of OP-B on NCI-H460, A549, A549/PTX and A549/DDP cells, with a concentration range of 1.25~20 μM for 24 or 48 h treatment (Fig. 1C). The results suggested that among the four kinds of cells, A549/DDP was more sensitive to OP-B, the effective concentration was 2.5 μM. More importantly, further combined action of OP-B and DDP (160 μM) on A549 and A549/DDP cells for 24 h verified that they had synergistic effect (Fig. 1D). Taken together, OP-B not only had significant inhibition effect on A549/DDP cells, but also increased the sensitivity of A549/DDP cells to DDP.

In order to determine whether OP-B has the same effect *in vivo*, we established the orthotopic xenograft lung cancer model and administrated the mice with 1.5 mg/kg or 3 mg/kg of OP-B for 25 consecutive days. The results showed that under the treatment of OP-B, mice weight between A549 and A549/DDP (Fig. 1E) groups was basically the same. Meanwhile, the tumor volume of both A549 and A549/DDP (Fig. 1F and G) groups decreased in dose and time dependent manner under the treatment of OP-B, and the inhibition rate on A549/DDP group was more significant than that on A549 (Fig. 1H).

### Inhibitory effect of OP-B on A549/DDP xenografted tumors in mice and zebrafish

**Figure 2:**
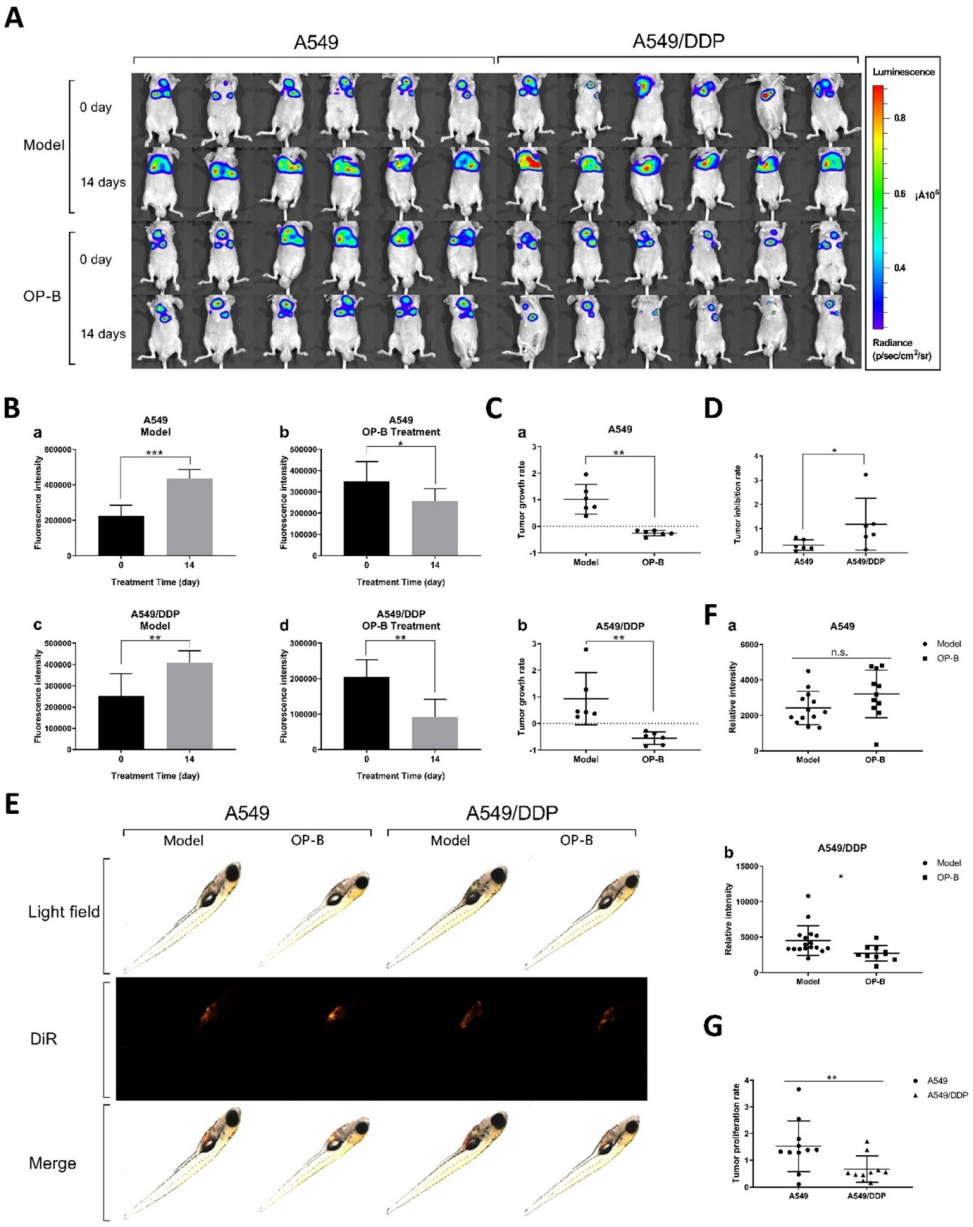
** OP-B also had a significant inhibitory effect on A549/DDP carcinoma *in situ* in mice and transplanted tumors in zebrafish. A.** Bioluminescent imaging and quantification of photon flux of 3 mg/kg OP-B treated groups with right lung parenchyma injection of luciferase-marked A549 and A549/DDP cells. **B.** Before and after OP-B treatment, the fluorescence intensity changes on model and OP-B groups of A549 and A549/DDP. **C.** Tumor growth rate of A549 (a) and A549/DDP (b) calculated according to the fluorescence intensity data. **D.** Comparison of tumor growth rates between A549 and A549/DDP. **E.** Representative images taken by fluorescence stereomicroscope of the zebra fish that were injected with A549 and A549/DDP cells and treated with 5 μM OP-B for 3 days. **F.** Relative fluorescence intensity of A549 (a) and A549/DDP (b) tumor in zebra fish after OP-B treatment. **G.** Comparison of tumor growth rate between A549 and A549/DDP that calculated according to the fluorescence intensity data. The bars and error bars indicate the mean ± SD. *p < 0.05, **p < 0.01, ***p < 0.005, ****p < 0.001

In order to verify whether OP-B has the same effect on tumor *in situ*, we inoculated A549 and A549/DDP cells on the right lung lobe of BALB/c nude mice (n=30), and observed tumor growth by bioluminescence image. After the models being successfully built, 3 mg/kg of OP-B was administered to the mice consecutively for 14 days, meanwhile, bioluminescence images were taken on 0 and 14th days (Fig. 2A). Statistical results showed that the fluorescence intensity in A549 and A549/DDP tumor-*in-situ* was significantly decreased by OP-B treatment (Fig. 2A and C), while between A549 and A549/DDP groups, OP-B inhibited A549/DDP tumor-*in-situ* more significantly than that in A549 groups (Fig. 2D).

In addition, we verified the effect of OP-B on proliferation of A549 and A549/DDP cells in zebra fish. To establish tumor model, A549 and A549/DDP cells labeled with DiR fluorescence were injected into the yolk sac (n=100) by microinjection technology, after 3 days treatment of 5 μM OP-B, the fluorescence intensity of zebrafish in each group was measured by fluorescent stereo-microscope (Fig. 2E). Statistics showed that the optical density of the A549 and A549/DDP (Fig. 2F) administration groups was significantly lower than that of the model groups. Similar to the results in mice, proliferation rate of the xenografted tumor in A549/DDP groups was significantly lower than that in A549 groups (Fig. 2G).

Taken together, OP-B has more significant inhibitory effect on DDP-resistant A549 tumors *in vivo* and *in vitro.*

### OP-B caused significant pyroptosis in A549/DDP cells

**Figure 3:**
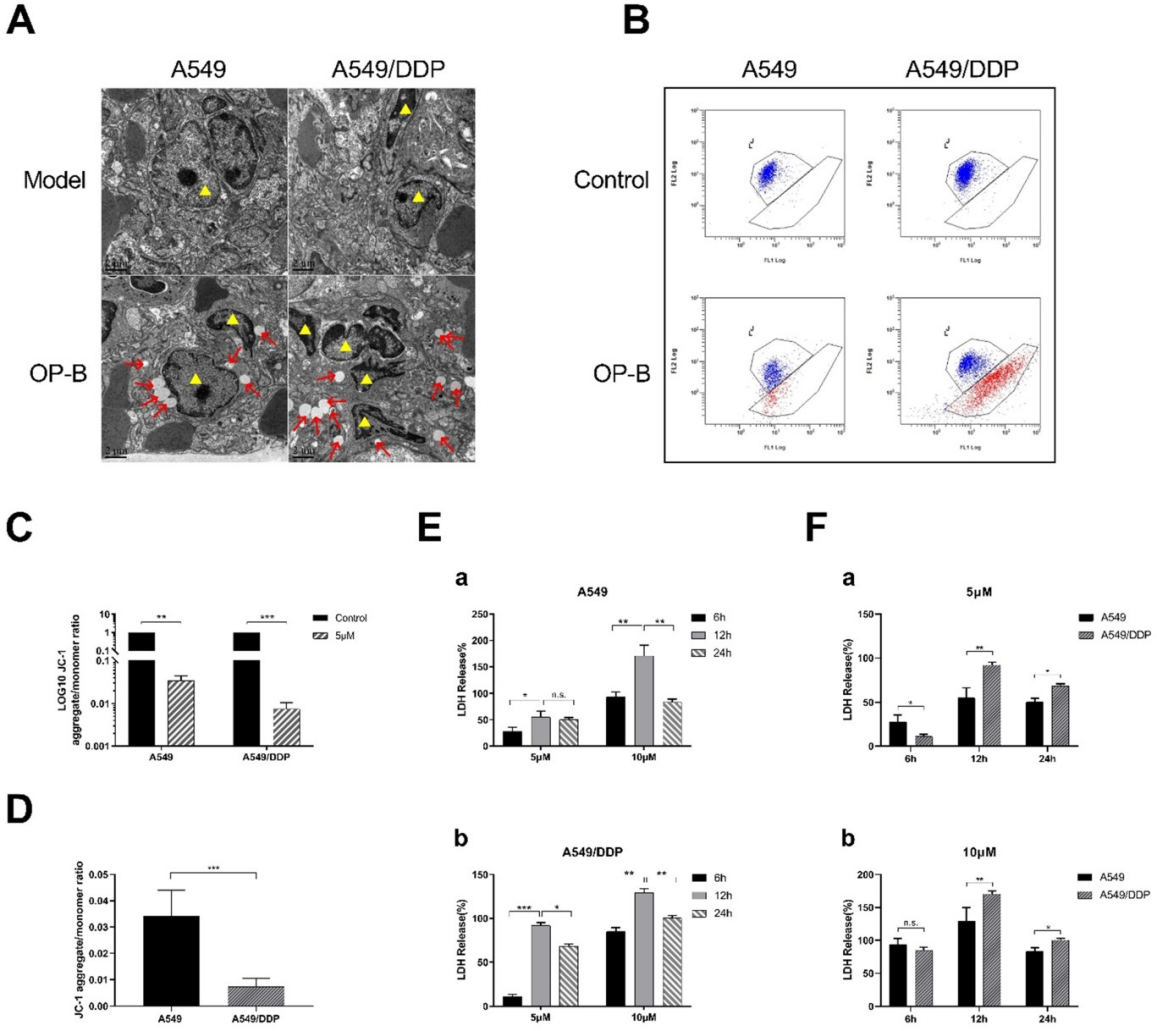
** OP-B caused significant pyroptosis in A549/DDP cells. A.** Transmission electron microscope images of tissue of tumor *in situ* (A549 and A549/DDP) after 3 mg/kg OP-B treatment for 14 days (Yellow triangles represent tumor cells). **B-D.** Mitochondrial membrane potential of A549 and A549/DDP cells was detected by flow cytometry after 5 μM OP-B treatment for 24 h. **E-F.** The level of LDH release in A549 (a) and A549/DDP (b) cells treated with different concentrations of OP-B after 6 h,12 h and 24 h.The bars and error bars indicate the mean ± SD. *p < 0.05, **p < 0.01, ***p < 0.005, ****p < 0.001

In order to further explore the reason why A549/DDP cells are more sensitive to OP-B than A549 cells, we took the lung tissues from the mice mentioned in Fig. 3A for transmission electron microscope (TEM) observation. We found that different degrees of swelling, accompanied by the rupture of the cell plasma membrane and the formation of bubble-like protrusions occurred in A549 and A549/DDP cells that infiltrated in lung tissue (Fig. 3A). We speculated that the reason why OP-B significantly inhibited the growth of A549/DDP cells compared with A549 cells, which due to more significant pyroptosis of A549/DDP cells.

Next, we used JC-1 mitochondrial membrane potential (MMP) probe to label the above cells. After 24 hours treatment with OP-B (5 μM), flow cytometry was used to detect the changes in the number of red and green fluorescence cells to quantify the MMP. The results showed that the level of MMP in A549 and A549/DDP cells decreased after OP-B treatment, while it decreased more significantly in A549/DDP cells (Fig. 3B-D).

Since the occurrence of pyroptosis is accompanied by the release of a large amount of inflammatory substance and decrease of mitochondrial membrane potential, we then tested LDH release rate and the changes of mitochondrial membrane potential (MMP) in A549 and A549/DDP cells. The results showed that after OP-B (5 μM and 10 μM) treatment for 6, 12 and 24 hours, the release of LDH in both cell lines increased in dose-dependent manner, and it reached peak at 12 h (Fig. 3E and F). Among them, the LDH release of A549/DDP cells was significantly higher than that of A549 cells.

### OP-B induced obvious pyroptosis in A549/DDP cancer *in vitro* and *in vivo*

**Figure 4:**
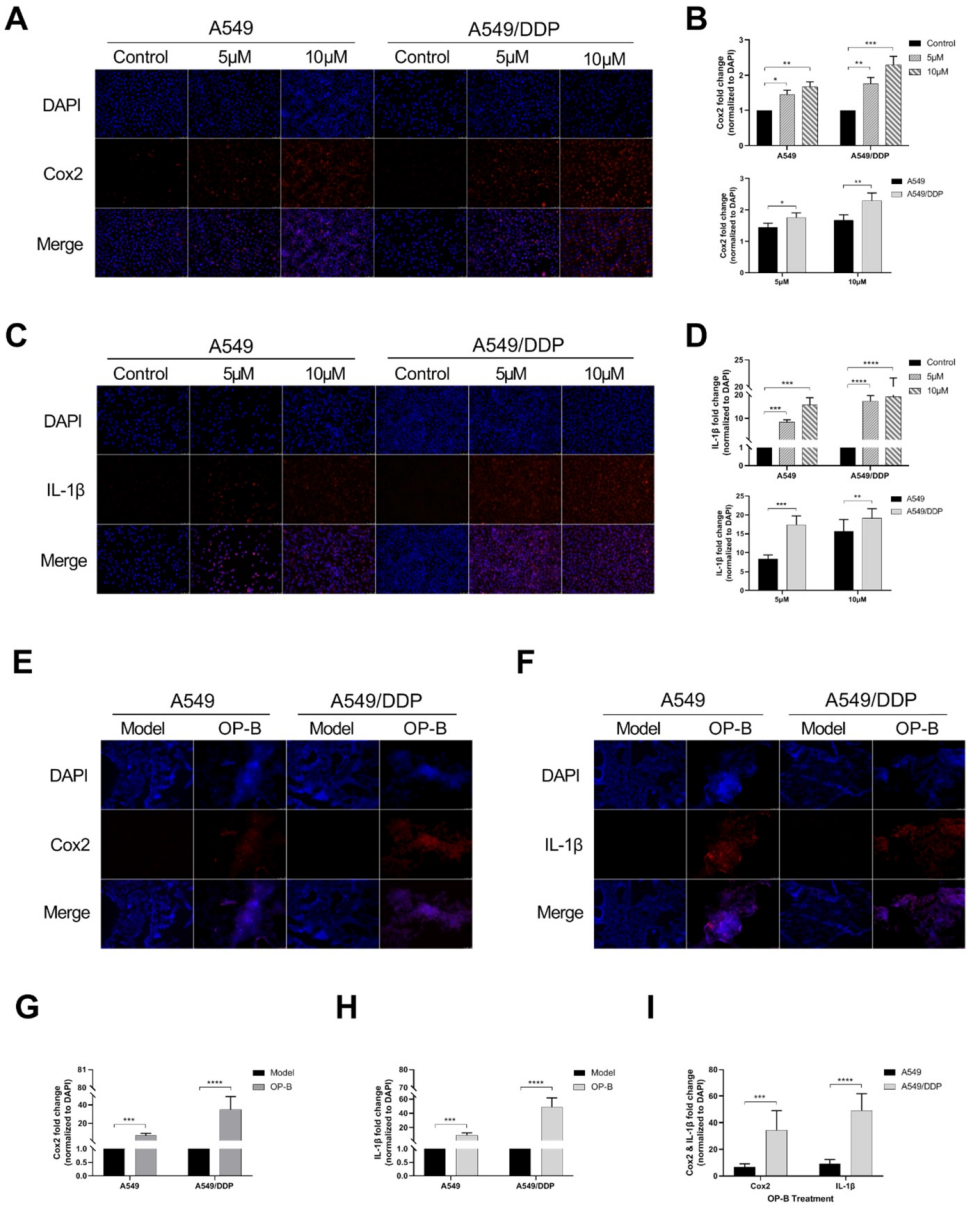
** OP-B induced obvious pyroptosis in A549/DDP cancer *in vitro* and *in vivo*. A.** Immunofluorescence staining of Cox2 expression in A549 and A549/DDP cells with different doses of OP-B treatment. **B.** Cox2 expression in A549 and A549/DDP cells treated with different concentration of OP-B. **C.** Immunofluorescence staining of IL-1β expression in A549 and A549/DDP cells with different concentrations of OP-B treatment. **D.** IL-1β expression in A549 and A549/DDP cells treated with different concentrations of OP-B. **E.** Immunofluorescence staining of Cox2 expression in different types of tumors *in situ*. **F.** Immunofluorescence staining of IL-1β expression in different types of tumors *in situ*. **G.** Cox2 expression in different types of tumors *in situ*. h IL-1β expression in different types of tumors *in situ*. i IL-1β and Cox2 expression in different types of tumors *in situ*. The bars and error bars indicate the mean ± SD. *p < 0.05, **p < 0.01, ***p < 0.005, ****p < 0.001

Since the occurrence of pyroptosis is often accompanied by the release of inflammatory factors, we detected the levels of Cox2 and IL-1β in the cells. After OP-B treatment (5 μM and 10 μM) for 24h, the immunofluorescence results showed that the expression levels of Cox2 and IL-1β in both of the cell lines were significantly increased, and the expression level of the two proteins was higher at 10 μM of OP-B than that at 5 μM (Fig. 4A-C). From the statistical results, it was obvious that the expression level of Cox2 and IL-1β in A549/DDP cells was higher than that in A549 cells (Fig. 4B and D). Furthermore, the results of the above were also proved *in vivo* (Fig. 4E-I).

### Overview of the diferentially expressed genes between A549/DDP cells treated with or without OP-B

**Figure 5:**
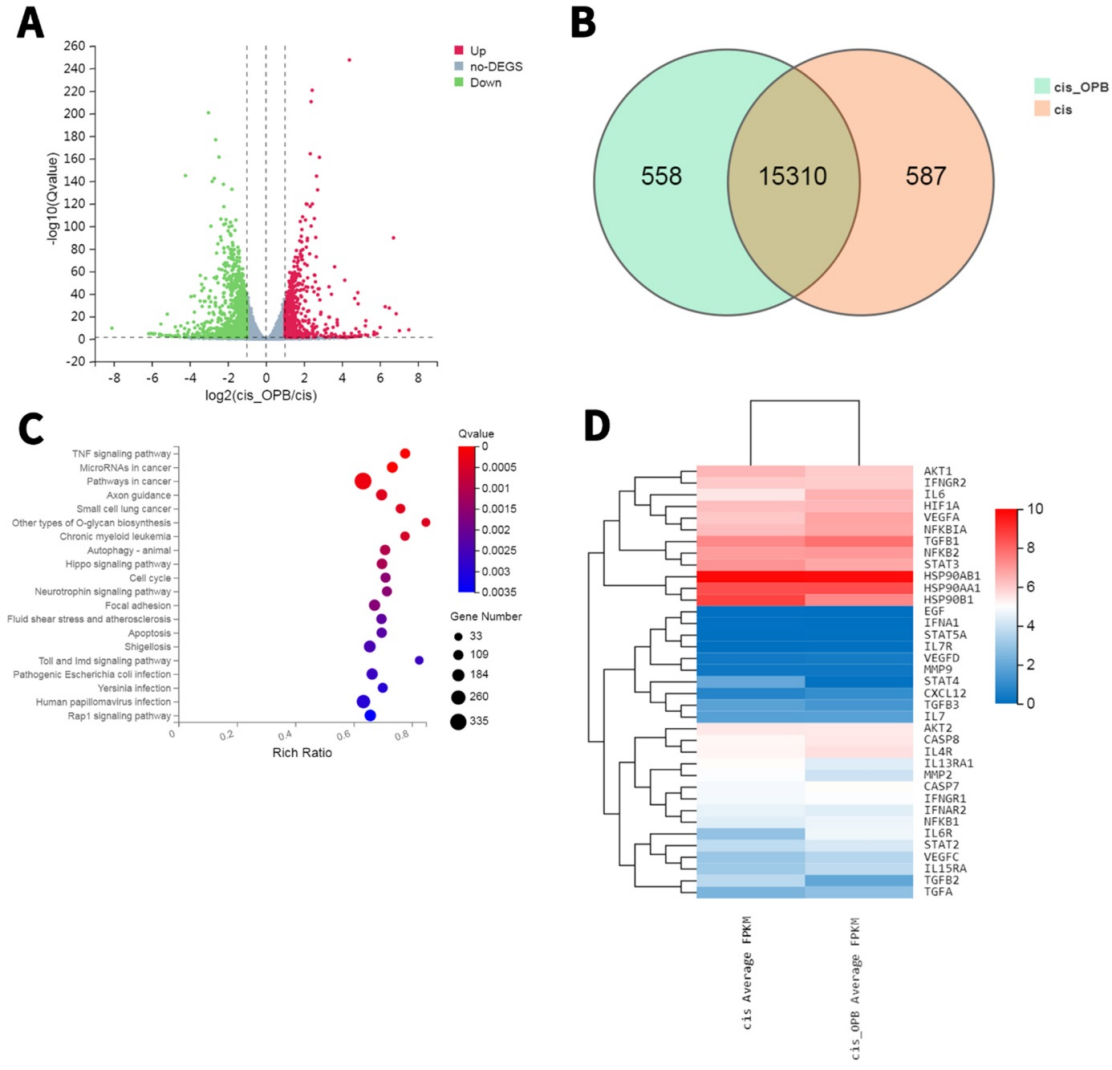
** RNA sequencing results reveal differentially expressed genes (DEGs) and enriched KEGG pathways in A549/DDP cells treated with or without OP-B. A.** Volcano plot (bottom) showing 690 decreased genes and 1149 upregulated genes. **B.** Venn diagram showing DEGs between A549/DDP cells treated with or without OP-B. **C.** Top enriched KEGG pathways for these 652 DEGs, including pyroptosis signaling. **D.** Transcriptiomic RNA-seq identified 1839 DEGs. Hierarchical cluster analysis (top) of significantly differentially expressed mRNAs between A549/DDP cells treated with or without OP-B.

To further elucidate the mechanism, we prepared RNA samples for transcriptomic RNA sequencing (RNA-seq) to screen differentially expressed genes (DEGs) between OP-B treated and the Mock A549/DDP cells. In a comparison between A549/DDP cells treated with or without OP-B, the transcriptome analysis found 1149 downregulated genes and 690 upregulated genes (Fig. 5A-D). The KEGG pathway enrichment of these DEGs showed that OP-B treatment significantly induced expression of proinflammatory cytokines IL-6 and pyroptosis related protein GSDMD, hinted that OP-B may regulate pyroptosis pathway, among the top 50 enriched pathways (with the top 35 enriched pathways shown in Fig. 5C-D).

### OP-B induced pyroptosis of A549/DDP cells by activating Caspase-1/GSDMD pathway

**Figure 6:**
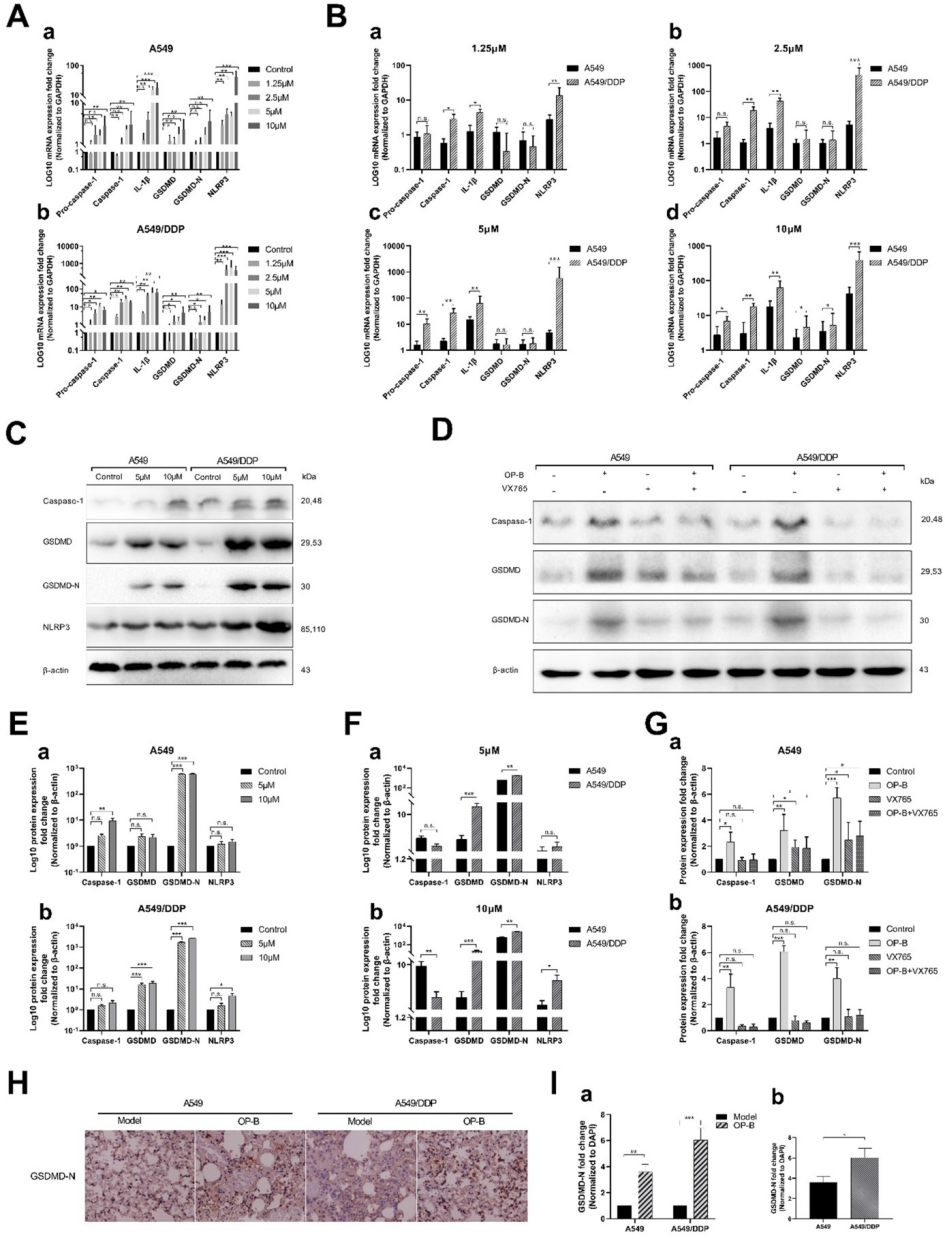
** OP-B induced pyroptosis of A549/DDP cells by activating Caspase-1/GSDMD pathway. A-B.** mRNA (pro-Caspase-1, Caspase-1, IL-1β, GSDMD, GSDMD-N and NLRP3) expression determined by qRT-PCR analysis in A549 and A549/DDP cells treated with different doses of OP-B. **C.** Protein (Caspase-1, GSDMD, GSDMD-N, NLRP3 and β-actin) expression determined by western blot analysis in A549 and A549/DDP cells treated with different concentrations of OP-B. **D.** Protein (Caspase-1, GSDMD, GSDMD-N and β-actin) expression determined by western blot analysis in A549 and A549/DDP cells treated with the inhibitor of VX765.**E-F.** Four kinds of protein expression level in A549 and A549/DDP cells treated with different concentrations of OP-B. **G.** Three kinds of protein expression level in A549 (a) and A549/DDP (b) cells treated with inhibitor of VX765. **H.** Representative images showing haematoxylin and eosin staining of lung samples from the different groups. **I.** GSDMD-N expression in the tissue of lungs of the different groups. The bars and error bars indicate the mean ± SD. *p < 0.05, **p < 0.01, ***p < 0.005, ****p < 0.001

In order to further explore the mechanism of OP-B-induced pyroptosis in A549/DDP cells, we detected the related mRNA expression levels of the classic pyroptosis pathway in A549 and A549/DDP cells. After different concentrations of OP-B acted on the two cells for 24 hours, qRT-PCR results showed that the expression level of pyroptosis-related mRNA increased in both of cells. We found that 2.5 μM of OP-B significantly upregulated pyroptosis-related mRNA only in A549/DDP cells, while in A549 cells, the concentration of OP-B needs to reach 5 μM that can upregulate mRNA. It suggested that A549/DDP cells were more sensitive to OP-B than A549 cells (Fig. 6A). Under the treatment of different concentration, the expression level of pyroptosis-related mRNA in A549/DDP cells was higher than that in A549 cells. It further showed that A549/DDP cells had a higher degree of pyroptosis than A549 cells (Fig. 6B).

In addition, we also tested the expression of related proteins in the classical pathway of pyroptosis under the action of OP-B (Fig. 6C). And to investigate whether Caspase-1 play a vital role in the pyroptosis of A549/DDP cells, the expression of related proteins was detected after adding Caspase-1 inhibitor VX765 (Fig. 6D). We found that under the action of 10 μM OP-B, the expression of pyroptosis-related proteins in A549 and A549/DDP cells was significantly up-regulated (Fig. 6E), while pyroptosis-related proteins in A549/DDP cells were up-regulated more significantly than that in A549 cells (Fig. 6F). After adding the inhibitor VX765, the downstream protein GSDMD-N of the pyroptosis pathway still expressed under the action of OP-B in A549 cells, while in A549/DDP cells it was inhibited (Fig. 6G), which suggested that in A549/DDP cells, the classic pyroptosis pathway of Caspase-1/GSDMD is the main pyroptosis mechanism.

Finally, we detected the expression of the key protein GSDMD-N in A549 and A549/DDP lung tissue of tumor *in situ*. The immunocytochemistry results further confirmed that OP-B could induce more obvious pyroptosis in A549/DDP tumor *in situ* (Fig. 6H and I).

## Discussion

Currently, the clinically used comprehensive treatment mainly based on chemotherapy, while the efficacy is not satisfactory. The main reason of it is the emergence of tumor multidrug resistance (MDR) 21. Therefore, it is very urgent to investigate the mechanism of MDR in lung cancer and find out the effective reversal strategy to solve it. Traditional Chinese Medicine has been paid more and more attention in MDR research of lung cancer due to the advantages of high efficiency, low toxicity and multi-targets.

Radix Ophiopogonis is commonly used in traditional Chinese medicine to treat lung diseases. In various chemical compositions of ophiopogonis, saponin is the key to quality control of it. OP-B, as a saponin compound, had been proved to have obvious anti-lung cancer effect *in vivo* and *in vitro* 17-19. However, whether it has the function of reversing drug resistance of lung cancer cells remains unknown. Herein, through a large number of cell and animal (mice and zebrafish) experiments, we verified that OP-B had a significant inhibitory effect on DDP resistant A549 cells. Meanwhile, in combination with DDP, OP-B enhanced the sensitivity of A549/DDP cells to DDP.

Further observation of the tumor tissues under electron microscope found that the morphology of the tumor cells in A549/DDP orthotopic tumor models showed more significant feature of pyroptotic cell death as bubbles swelling from the plasma membrane and causing lysis and massive release of cellular contents. As occurrence of pyroptosis is always followed by releasing of many proinflammatory factors, including IL-1β, IL-18, ATP, and HMGB1 22-24 and excessive Caspase activation 23. Through transcriptomic sequencing we found that OP-B induced more significant release of IL-6 in OP-B treated A549/DDP group than that in the Mock A549/DDP group. Moreover, from the gene sequencing database we screened out GSDMD to be the more significant differentially expressed gene. Further detection of pyroptosis related indexes, such as LDH, MMP, Cox2, IL-1β *in vivo* and *in vitro* also verified the hypothesis of pyroptosis, and further investigation of Caspase-1/GSDMD pathway by qRT-PCR and western blot both proved that OP-B induced more significant pyroptosis in A549/DDP cells than that in A549 cells.

As tumor cells always show innate resistance to apoptosis, the development of new strategies to induce pyroptosis may provide more efficient cancer therapy options and improve patient survival 25, 26. Two recently published studies both reported that tumor cells undergoing pyroptosis resulted in recruitment of tumor-suppressed immune cells ^23, 24^. In these two works, Zhang *et al*. found that in the pyroptosis-activated immune microenvironment, CD8+ T cells and NK cells reciprocally induced pyroptosis in tumor cells via granzyme B, thereby establishing a positive feedback loop 27. Wang *et al*. revealed that pyroptosis by their bioorthogonal system in less than 15% of tumor cells was sufficient to clear an entire tumor graft operating in live animals 28. So, it is very interesting and worthy of further investigating whether OP-B induced pyroptosis in A549/DDP lung cancer cells could also lead to immune-activation.

**Figure 7:**
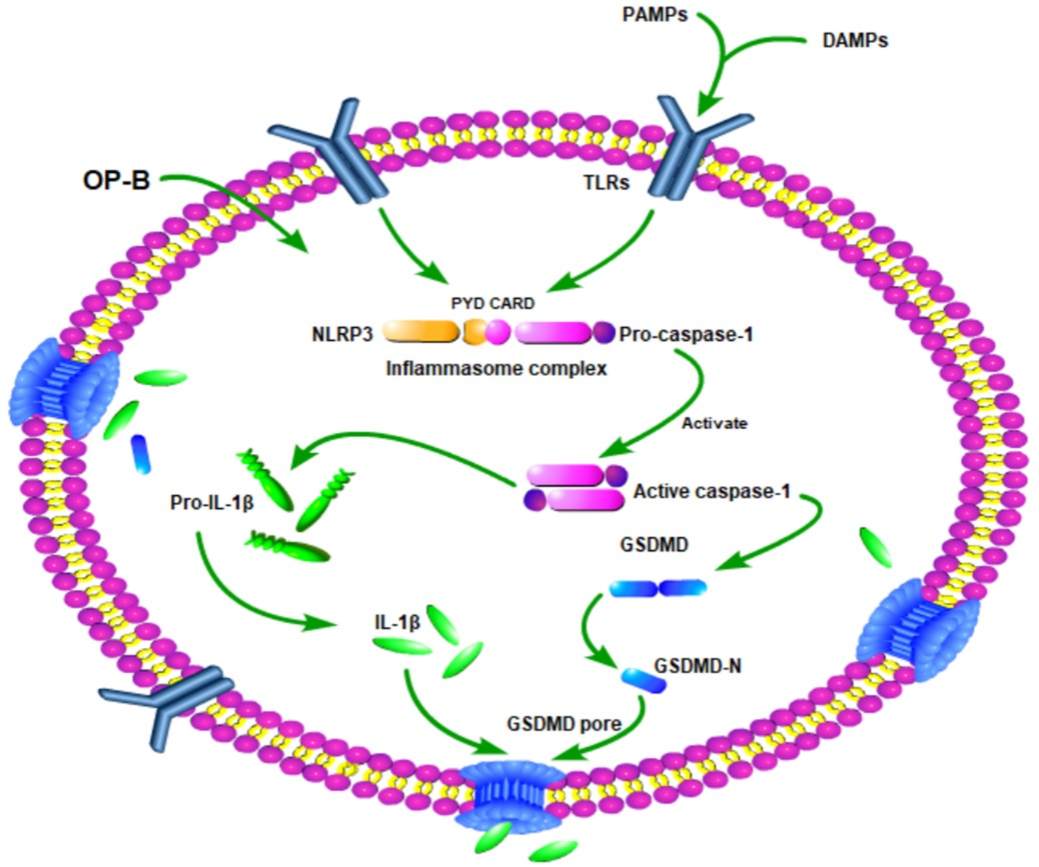
Schematic diagram of mechanism of this research. OP-B induced A549/DDP cells pyroptosis by Caspase-1/GSDMD signal pathway.

In summary, our study demonstrated that OP-B suppressed A549/DDP cells growth more significantly than A549 cells. The mechanism of it may involve that OP-B induced A549/DDP cells produced more significantly pyroptosis than A549 cells by Caspase-1/GSDMD pyroptosis pathway (Fig. 7). Our finding is helpful to provide new insights into the mechanism of drug-resistant cell death and provide new ideas and directions for the clinical treatment of drug-resistant tumors.
